# Mixed methods to evaluate knowledge, attitudes and practices (KAP) towards rabies in central and remote communities of Moramanga district, Madagascar

**DOI:** 10.1371/journal.pntd.0012064

**Published:** 2024-03-29

**Authors:** Claire Leblanc, Daouda Kassié, Mendrika Ranaivoharimina, Elliot Fara Nandrasana Rakotomanana, Reziky Tiandraza Mangahasimbola, Anjasoa Randrianarijaona, Ravo Ramiandrasoa, Alphonse José Nely, Nivohanitra Perle Razafindraibe, Soa Fy Andriamandimby, Dany Bakoly Ranoaritiana, Virginie Rajaonarivony, Laurence Randrianasolo, Laurence Baril, Chiarella Mattern, Rila Ratovoson, Hélène Guis

**Affiliations:** 1 Epidemiology and Clinical Research Unit, Institut Pasteur de Madagascar, Antananarivo, Madagascar; 2 General Paediatrics and Paediatric Infectious Disease Unit, Nantes University Hospital, Nantes, France; 3 CIRAD, UMR ASTRE, Antananarivo, Madagascar; 4 ASTRE, Univ Montpellier, CIRAD, INRAE, Montpellier, France; 5 Vaccination Center, Institut Pasteur de Madagascar, Antananarivo, Madagascar; 6 Service for the Fight against Plague, Emerging and Re-emerging Diseases and Neglected Tropical Endemo-Epidemic Diseases, Ministry of Public Health, Antananarivo, Madagascar; 7 WHO Madagascar, Antananarivo 101, Madagascar; 8 Directorate of Veterinary Services, Ministry of Agriculture and Livestock, Antananarivo, Madagascar; 9 National Laboratory of Rabies, Virology Unit, Institut Pasteur de Madagascar, Antananarivo, Madagascar; 10 Direction of Health Monitoring, Epidemiological Surveillance and Response (DVSSER), Ministry of Public Health, Antananarivo, Madagascar; 11 Institut Pasteur du Cambodge, Phnom Penh, Cambodia; 12 Ceped (Institut de Recherche pour le Développement, Université de Paris, INSERM), Paris, France; University of California Berkeley, UNITED STATES

## Abstract

Control of dog-mediated rabies relies on raising awareness, access to post-exposure prophylaxis (PEP) and mass dog vaccination. To assess rabies awareness in Moramanga district, Madagascar, where rabies is endemic, two complementary quantitative and qualitative approaches were carried out in 2018. In the quantitative approach, a standardized questionnaire was administered to 334 randomized participants living in 170 households located less than 5 km from the anti-rabies treatment center (ARTC) located in Moramanga city (thereafter called the central area), and in 164 households located more than 15 km away from the ARTC in two rural communes (thereafter called the remote area). Logistic regression models were fitted to identify factors influencing knowledge and practice scores. The qualitative approach consisted in semi-structured interviews conducted with 28 bite victims who had consulted the ARTC, three owners of biting dogs, three ARTC staff and two local authorities.

Overall, 15.6% (52/334) of households owned at least one dog. The dog-to-human ratio was 1:17.6. The central area had a significantly higher dog bite incidence (0.53 per 100 person-years, 95% CI: 0.31–0.85) compared to the remote area (0.22 per 100 person-years, 95% CI: 0.09–0.43) (p = 0.03). The care pathway following a bite depended on wound severity, how the dog was perceived and its owner’s willingness to cover costs. Rabies vaccination coverage in dogs in the remote area was extremely low (2.4%). Respondents knew that vaccination prevented animal rabies but owners considered that their own dogs were harmless and cited access and cost of vaccine as main barriers. Most respondents were not aware of the existence of the ARTC (85.3%), did not know the importance of timely access to PEP (92.2%) or that biting dogs should be isolated (89.5%) and monitored. Good knowledge scores were significantly associated with having a higher socio-economic status (OR = 2.08, CI = 1.33–3.26) and living in central area (OR = 1.91, CI = 1.22–3.00). Good practice scores were significantly associated with living in central area (OR = 4.78, CI = 2.98–7.77) and being aware of the ARTC’s existence (OR = 2.29, CI = 1.14–4.80).

In Madagascar, knowledge on rabies was disparate with important gaps on PEP and animal management. Awareness campaigns should inform communities (i) on the importance of seeking PEP as soon as possible after an exposure, whatever the severity of the wound and the type of biting dog who caused it, and (ii) on the existence and location of ARTCs where free-of-charge PEP is available. They should also encourage owners to isolate and monitor the health of biting dogs. Above all, awareness and dog vaccination campaigns should be designed so as to reach the more vulnerable remote rural populations as knowledge, good practices and vaccination coverage were lower in these areas. They should also target households with a lower socio-economic status. If awareness campaigns are likely to succeed in improving access to ARTCs in Madagascar, their impact on prompting dog owners to vaccinate their own dogs seems more uncertain given the financial and access barriers. Therefore, to reach the 70% dog vaccination coverage goal targeted in rabies elimination programs, awareness campaigns must be combined with free-of-charge mass dog vaccination.

## Author summary

Despite effective animal and human vaccines, rabies causes over 59,000 human deaths annually, mainly in children and in poor rural communities in Asia and Africa. World Health Organization and partners have recently launched the “Zero by 30” global plan which aims to end human deaths by dog-mediated rabies by 2030 by raising awareness, increasing access to post-exposure prophylaxis (PEP) and mass dog vaccination.

In Madagascar, where rabies is endemic, a network of anti-rabies treatment centers (ARTC) delivers free-of-charge vaccines to exposed patients. As little is known on rabies awareness in Malagasy communities, we set up quantitative and qualitative surveys on rabies knowledge, attitudes and practices (KAP).

Results showed that major hosts, main transmission routes, clinical signs and outcomes were known by most respondents. Yet knowledge on the existence of ARTC, the usefulness and availability of PEP, usefulness of confining and monitoring biting dogs was very low. Although bite incidence was lower, remote rural areas had more limited knowledge, poorer practices on rabies and extremely low dog vaccination coverage, so awareness campaigns should make sure remote rural populations are reached. If these campaigns are likely to succeed in improving access to ARTCs, their impact on dog vaccination seems more uncertain. Therefore, to reach dog vaccination coverage goals, the center-piece of rabies elimination strategies, awareness campaigns must be combined with free-of-charge mass dog vaccination.

## Introduction

Rabies is considered by the World Health Organization (WHO) as a neglected disease which causes over 59,000 human deaths per year throughout the world in more than 150 countries [1,2]. Most deaths occur in Asia and Africa, in poor rural communities. Half of the world notified cases are children under 15 years of age [1,3]. This acute, progressive and invariably fatal encephalomyelitis, is caused by viruses belonging to the *Lyssavirus* genus (*Mononegavirales* order, *Rhabdoviridae* family) [4] which are transmitted through the saliva of infected animals. Among the *Lyssavirus* genus, *Lyssavirus rabies* species (RABV) is essentially transmitted by carnivores, in particular domestic dogs and a variety of bat species, and is by far the most frequent Lyssavirus infection in humans. In 99% of cases, transmission of RABV to humans occurs through bites of infected dogs [3]. Rabies is described as "100% preventable" with the existence of effective vaccines for animals and humans, but also "100% lethal" after the onset of clinical signs [5]. The global financial burden of rabies is estimated to be US$124 billion annually, with 45% of this burden falling on Africa, where human mortality due to rabies is particularly high [6]. The fact that dogs have no or very little economic value and the paramount need for integrated One Health response contribute to the underreporting and neglect of rabies [7,8]. In 2015, WHO, the World Organization for Animal Health (WOAH, ex-OIE), the Food and Agriculture Organization of the United Nations (FAO) and the Global Alliance for Rabies Control (GARC) developed a global strategic plan to eliminate dog-mediated human rabies by 2030 (Zero by 30) [2]. To reduce human rabies risk, this plan relies on three main pillars: raising awareness of the population on rabies, access to PEP and mass dog vaccination [2].

In Madagascar, rabies is endemic and is a major public health problem. Each year, three to 10 human cases and about 50 to 60 animal rabies cases are confirmed by the National Reference Laboratory (LNR) [9,10]. These results probably reflect only the tip of the iceberg [10,11], partly because of low awareness towards rabies, poor surveillance, combined with difficulties to access anti-rabies treatment centers [12] and because of difficulties to sample suspect human and animal cases (infected people often die at home, biting animals may be far away, buried, sold, eaten or hard to find, lack of funding to reach animal and human suspect cases, lack of training and material to sample, non-vaccination of people in charge of sampling) and ship samples from remote rural areas [10,11,13]. Indeed, a recent study has estimated an annual incidence of 960 (790 to 1120) human deaths per year due to rabies in Madagascar with the current level of PEP avoiding a further 800 (640–970) deaths per year [12], globally in line with previous estimates [14].

PEP was first delivered in Madagascar in 1898 when Institut Pasteur de Madagascar (IPM) set up the first anti-rabies treatment center (ARTC) in Antananarivo, the capital city. This ARTC is still active and delivers PEP to 6,000 patients per year on average [15]. To increase access to PEP, the Ministry of Public Health has set up 30 other ARTCs distributed throughout the country (in all 22 regions) [9,10,15,16]. Vaccines (VERORAB vaccine, Sanofi Pasteur, Marcy l’Etoile, France) and syringes are provided to ARTCs free of charge by IPM. In turn, PEP or at least the vaccine is free of charge for exposed patients. Madagascar is thus one of the rare sub-Saharan African countries where rabies PEP is free and theoretically accessible at the regional level for the entire country [17]. Yet geographic access to resources in Madagascar is challenging: the country has one of the least developed road networks of the world with 5.4 km of roads per 100 km^2^ of land and only 11.4% of the population living less than 2 km away from an all-season road [18]. Access to health care is a major problem, including at very local scales such as the access to community health workers. Indeed, a recent study showed that the consultation rate of community health workers was reduced by 28.1% for each increase of 1 km in the distance with the patient’s house [19]. Similarly, access to PEP remains a major issue in Madagascar [12]. Indeed, spatial modelling of access to PEP in Madagascar showed that travel times to ARTCs significantly increased rabies death incidence and thus that most deaths occurred in communities with the least access to ARTCs [12]. The Malagasy national PEP protocol follows the 2018 WHO guidelines and is known as abridged or updated Thai Red Cross protocol or the Institut Pasteur du Cambodge regimen (*i*.*e*., the 3-dose intradermal 1 week protocol) [20]. Rabies immunoglobulins are only available at the ARTC located at IPM in the capital.

In African and Asian rabies endemic countries, rabies virus is transmitted to humans mainly by dog bites [20]. Dogs in Madagascar resemble pariah dogs [21] and genetic analyses suggest that contrary to human settlement, they originated entirely in Africa [22]. Their relation with man and how they are perceived today in Madagascar has been documented in the vicinity of two conservation areas and results show that i) most owned dogs can roam freely part or all the day, ii) most are kept for protection purposes (cited by more than 80% of owners) and, to a lesser extent, for companionship (18.5–26.5%), iii) more than 50% of both dog owners and non-dog owners had a negative perception of free-roaming dogs, and iv) the vast majority of people approve of spay/neuter/vaccine programs and state that they would use them if they were freely available [23,24].

Dog bite incidence estimates in Africa can vary greatly depending on multiple factors including dog and human densities and demographic characteristics, dog-human relations (interaction type and frequency) and social, economic and environmental settings. In Democratic Republic of Congo, 5 bites per 1,000 person-years were recorded [25]. An annual bite incidence of 2.6% was recorded in Cameroon [26] whereas in Tanzania it reached 8% [27]. In Ethiopia, 14.1% of households reported having had a member bitten in the past [28]. Another study in Ethiopia reported 0/471 of people living in an urban area and 9/49 (18.4%) pastoralists had a member of their household bitten in the last 5 years [29]. In a study carried out in 2007 in Antananarivo city, the capital of Madagascar, 5.4% and 11.1% of households of the 1^st^ and 6^th^ borough (arrondissement) respectively declared that at least one bite incident had occurred among the members of their family [30]. In the provincial city of Moramanga (Madagascar), in 2010, bite incidents among a member of the household were reported in 20.6% of households [8]. In the two latter studies carried out in Madagascar, the timing of the bite incident and the precise denominator were not available, so precise estimates of bite incidence per person were not calculated.

In Madagascar, dog owners can use either imported inactivated rabies vaccines or locally-produced live attenuated rabies vaccines (Lyorab). A study evaluating dog vaccination coverage in Antananarivo in 2007–2008 showed that the percentage of dogs declared by owners as regularly vaccinated against rabies was 21.6% (95% confidence interval (CI): [20.0%– 23.4%]) but the percentage dropped to 7.2% (95% CI: [6.2%– 8.4%] when considering those with a valid vaccination certificate [31]. Similarly, in Moramanga city commune, in 2012, 37.0% (111/300) of dog owning households declared vaccinating their dogs, but only 11.7% (35/300) had a valid vaccination certificate [8], suggesting that most dogs did not receive the recommended annual boosters. In rural areas, in the absence of mass dog vaccination campaigns, the percentage of vaccinated dogs probably does not exceed 5% [32]. Mass dog vaccination campaigns have been set up occasionally in a few localities by non-governmental organizations such as Mad Dog Initiative [23,32], and in 2019 and 2020, by the government in Analamanga region and around Ambatondrazaka (Alaotra-Mangoro region), but the latter campaign was hampered by the COVID-19 pandemic and other logistic issues [10]. The law in Madagascar stipulates that a dog that has bitten a human must be observed by a veterinarian three times during the following 15 days. In practice the law is rarely enforced [10] although the ARTCs have the possibility to request the police if the dog owner does not comply with regulations.

Although there are a growing number of studies assessing knowledge, attitudes and practices (KAP) towards rabies in Africa [25–29,33–46] and elsewhere ([47–57]), to our knowledge, the few studies on knowledge, attitudes and/or practices towards rabies carried out in Madagascar [8,58] have not been published in scientific journals. One of these studies targeted only dog owners (n = 96), from Antananarivo, in 2015, and showed that owners who owned pedigree dogs had better knowledge and practices and that knowledge impacted practices [58]. In this study, no socio-economic parameters on the participants were included. Among notable results, 42 (43,8%) of owners thought that their dogs were not at risk of rabies, and among them, 8 (19% of them) doubted that rabies existed, showing the need to increase awareness on rabies. Another notable result was that 88.5% of owners knew that there was an ARTC at IPM (the first ARTC created and the one with delivering PEP to the greatest number of patients). Whether the existence of the 30 more recent provincial ARTCs benefit from the same notoriety among the general population remains to be assessed. The second study consisted in a survey carried out in Moramanga in 2010 among 746 households showed that 23.2% of households owned at least one dog at the time of the study, 75.4% of participants knew that rabies could be transmitted through the bite of an infected animal but only 19.1% knew that rabies was not curable after symptom onset. Unfortunately, only global scores encompassing answers to several questions on knowledge or practices were reported and not precise answers to specific questions, making it more difficult to identify messages to target in awareness campaigns. Although 5.3% of all individuals of the households (184/3444) had been bitten by a dog, data on dog bite could only be collected on 93 participants who were physically present at the time of the study (mainly women (65.6%)), and among these 93 bite victims, only 22 (23.7%) received a full PEP. Those who had not sought PEP justified this behavior because of the small size of the wound (51.5%), non-availability of the vaccine, the doctor or of the bite victim to go to seek PEP (43.9%) and the cost (4.6%), suggesting that these victims might not have been fully aware of what services were proposed in ARTC and how important it was to seek PEP.

So far, awareness-raising campaigns on rabies have been limited both in frequency and in extent. No national awareness campaign has been set up in the last decade. Some non-governmental organizations such as Mad Dog Initiative have set up awareness campaigns alongside to their vaccination and spay/neuter campaigns in two districts of the eastern coast [23]. Occasional initiatives are also set up to celebrate World Rabies Day (28^th^ September), but these are often limited to a few towns. Given the low level of education, the limited number of ARTCs and veterinarians, the dramatically poor quality and quantity of roads resulting in strong isolation and low access to human and animal healthcare [12], community knowledge on rabies is probably limited. The way dogs are perceived, their role and the human-dog relationship also impact perceptions and practices on rabies and need to be documented to better understand potential barriers to rabies control [21,22].

Assessing KAP towards rabies is essential to target communities and messages to deliver during awareness campaigns towards groups which need it the most and on elements which hinder correct practices. To this end, a two-pronged approach was carried out in Moramanga district, Madagascar: i) a quantitative approach on knowledge and practices towards rabies in households located within 5 km from Moramanga city’s ARTC and in more remote households located more than 15 km away from the ARTC and ii) a qualitative approach complementing the first approach, to better understand

- interactions between humans and dogs including attitudes towards dogs, dog bites and dog vaccination,
- biting dog management practices,
- and the care pathway after a bite in order to identify levers and obstacles to biomedical care seeking behavior.

## Methods

### Ethical considerations

The study was conducted in compliance with the principles set out by the Declaration of Helsinki, and the regulatory requirements of the Malagasy government on Health Research Ethics. The protocol of the quantitative approach was approved by the National Ethics Committee for Biomedical Research of Madagascar (N° 127-MSNP/CERBM, December 19^th^, 2017). A written consent was obtained from all adult respondents. Inclusion of adult respondents were favored, when possible, but if only minor respondents were available for the interview, a formal verbal consent was obtained from their parents or guardians. A specific protocol for the qualitative part of the study was approved by the National Ethics Committee for Biomedical Research of Madagascar (N° 129-MSANP/CERBM, October 23^rd^, 2018).

For both surveys, the field teams ensured that the interviewees received clear and precise information about the research process before starting the interviews. To this end, the objective and the study process, as well as its duration and modalities, were explained in detail to each participant. It was specified that participants were free to choose to participate or not and that each participant had the right to leave the interview at any time, without being obliged to give any explanation. To ensure the confidentiality of the participants and of the information gathered, (i) a code was attributed to each interviewee so that no-one could recognize them, and their names never appeared in any documents relating to this study and (ii) digitalized data was stored in a computer and protected with a password.

### Study area

**Fig 1:**
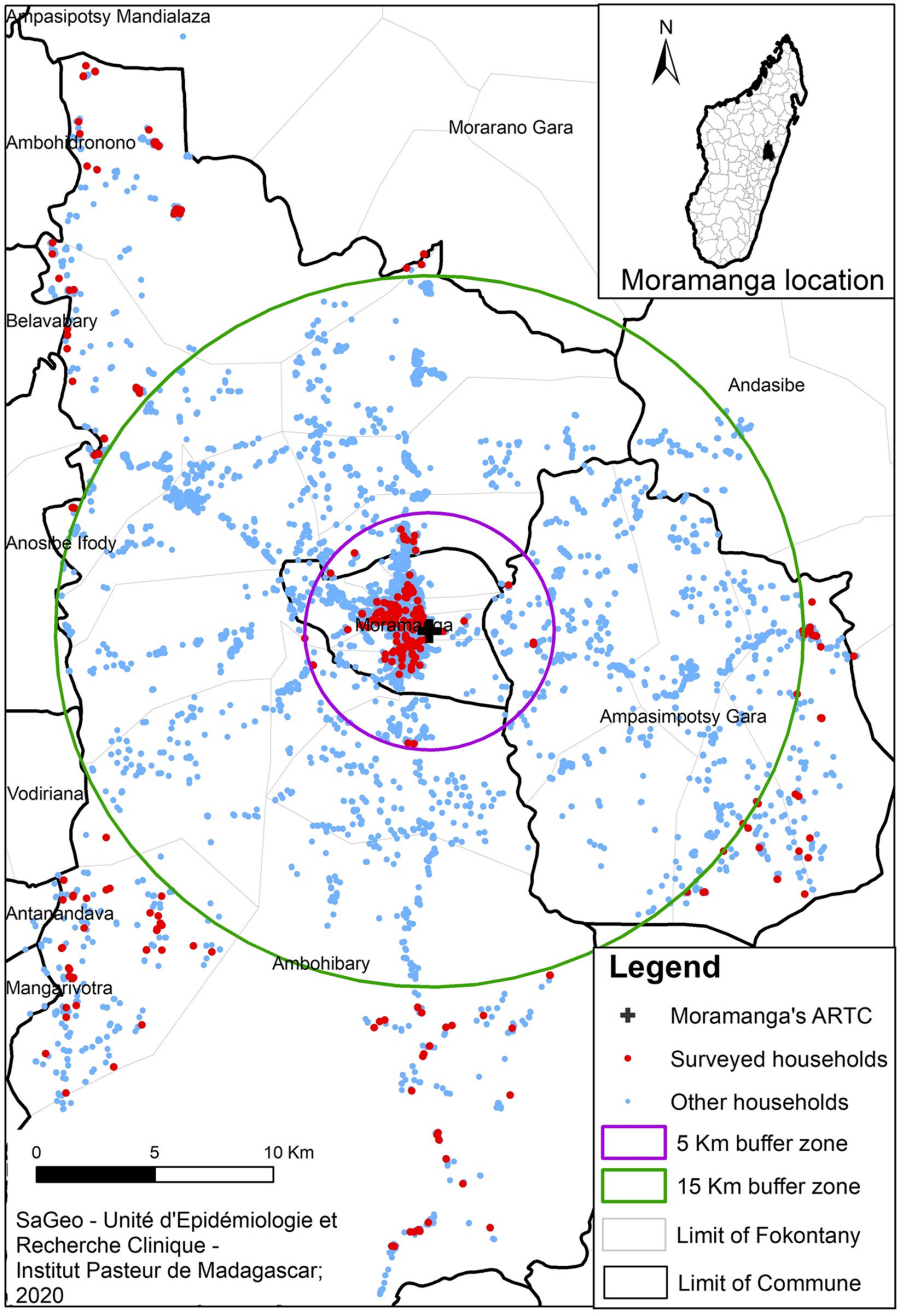
Map of the households in Moramanga district included in the quantitative study. Source of administrative boundaries: https://data.humdata.org/dataset/cod-ab-mdg.

Moramanga district is located in the middle-eastern part of Madagascar, in the Alaotra-Mangoro region, at the boundary between eastern tropical coastal areas and the more temperate central highlands, at an average altitude of 900 m above sea level (Fig 1). National census data recorded 350,724 inhabitants in 2018 in Moramanga district [59]. The main city of the district, Moramanga city, is a medium-sized provincial city [60], located on the national road 2 joining Antananarivo, the capital city (located at a road distance of 115 km) to Toamasina, the second most populated city and chief seaport of the country (located at a road distance of 239 km) and on national road 44 which leads to the most important rice-producing area of the country (Ambatondrazaka area). Yet Alaotra Mangoro region remains poor: in 2013, 82% of roads were earth roads, 76% of communes were not connected to the power grid and 78% did not have access to a water network [61]. In 2018, considering a global multidimensional poverty index (an index which reflects the deprivations faced in terms of education, health and living status), the incidence of poverty in the region was 68.0%, close to the national mean value of 70.3%, making it the 7^th^ least poor region of Madagascar (out of 22) [62].

Moramanga district encompasses a major industrial mine of nickel and cobalt (Ambatovy) [60] and the Andasibe National Park. Alongside to mining and tourism, the main economic activities of the district include timber exploitation, trade and agriculture [60]. Yet, a substantial proportion of population lives in poverty: in 2008, a survey conducted by the United Nations estimated that 65% of the households in Moramanga city lived in precarious or informal housing and that the average daily income per person was 0.45US$, under the poverty line [60].

### Community knowledge and practices quantitative approach

#### Sampling design

Moramanga district was chosen for both studies because i) it is an area where animal and human rabies are endemic [14], ii) there is an ARTC in Moramanga city, and iii) previous data collected within the Moramanga Health Survey in Urban and Rural areas in Madagascar (MHURAM project) [63] facilitated the description of the population in the study area.

The MHURAM project aims to systematically collect information on causes of death through verbal autopsies, filling an important gap as in Madagascar, the collection of this data is so far only carried out in major cities. Interestingly, it is the only cohort in Madagascar which covers both rural and urban communes. One of its specific aims is to establish baseline demographic, socio-economic, environmental and health data in three of the 21 communes of the district: Moramanga city commune, the main urban commune of the district, and two adjoining rural communes, Ambohibary and Ampasimpotsy. Two further characteristics make it an interesting study area: (i) the socio-demographic profile of its population is similar to the general population of Madagascar and (ii) local authorities are collaborative [63].

Given that this KAP study aimed to include households located close (<5 km) and further (>15 km) to the ARTC, the study area encompassed both the urban commune of Moramanga city and the two adjoining rural communes Ambohibary and Ampasimpotsy. MHURAM census data for 2014–2016 recorded 40,767 inhabitants in Moramanga city commune and 37,634 in the two rural communes [63]. In MHURAM study area, 63% of urban households had access to electricity in urban area against only about 7% in rural area [63]. In the urban commune of Moramanga, a previous study carried out in 2010 reported a mean dog-to-human ratio of 1:7.2 (*i*.*e*., a total of 4,990 dogs) [8].

A prospective cross-sectional study was carried out from the 30^th^ of October 2018 to the 22^nd^ of December 2018 in three communes in Moramanga district (Moramanga city, Ambohibary and Ampasimpotsy). We made the hypotheses that (i) most people living within Moramanga district would travel by foot to reach the ARTC given global poverty levels [62] and the scarcity and poor conditions of the road network in Madagascar [18] and that (ii) even a small distance of a few kilometers would impact KAP towards rabies. To test whether household distance to an ARTC impacted knowledge and practices, we arbitrarily chose to compare people living in households located within 5 km of Moramanga ARTC, located in the urban commune of Moramanga city or very close to it, thereafter called “central area” to people living in more remote rural households, defined as located beyond a radius of 15 km from the ARTC, thereafter called “remote area”. These more remote households were located in the two adjoining rural communes, Ambohibary and Ampasimpotsy (Fig 1). The choice of 5 km defining the central area corresponded to a distance which would take roughly 1 hour to cover by foot, whereas people living beyond 15 km, in the remote area, would need several hours to reach the Moramanga ARTC by foot.

A randomized sample of household members were identified and interviewed. The randomization was done from the households registered using a Global Positioning System (GPS) in the study area of the MHURAM project (Fig 1). This study was initially designed to provide some general information about dog bites in the study area. Dog bite incidence estimates in Africa can vary greatly: annual bite incidences of 0.5% to 8% were recorded in Democratic Republic of Congo [25] and Tanzania [27] respectively. A previous study in Moramanga city estimated that 20.6% of households had been bitten by a dog [8]. To be conservative, we estimated that in the three communes of the study area, 25% of households could have been bitten over the last five years as this would correspond to 1% of the household members being bitten each year under the hypotheses that households would comprise 5 individuals on average and that, in the households in which bites would occur, a single person per household would be bitten in the 5-year period. Using the formula for large population size *n* = *z*^2^ * *p*(1 − *p*)/*e*^2^, the sample size recommended for the estimation of a single proportion of p = 0.25 of households having experienced a bite over the last 5 years, with a margin of error of e = 0.05 and a level of confidence of 95% (z = 1,96) was 288 households. Considering a non-response rate of 10%, the aim was to investigate at least 317 households. The same number of households were investigated within 5 km radius of the Moramanga ARTC (central area) (n = 159 households) and within those located beyond a radius of 15 km (remote area) (n = 159 households).

#### Inclusion criteria

Inclusion criteria in the MHURAM project were living in the Moramanga district and not planning on moving out in the next 3 months. The inclusion criteria for the rabies quantitative study were household members already identified through the MHURAM project (*i*.*e*., corresponding to the inclusion criteria of MHURAM) and who agreed to sign an informed consent. Non-inclusion criteria were the household members that were absent for more than 6 months, the household members where no adults were present during the investigation or a refusal to participate.

#### Questionnaire

A standardized questionnaire was designed and pre-tested a first time with 5 people interviewed in Antananarivo, within Institut Pasteur of Madagascar (Antananarivo), and a second time with 8 people interviewed in Ambohitranjavidy and Tanambao, villages located in Moramanga city commune and 10 people living in Ankarahara, a village located in the rural commune of Ambohibary. This enabled to check its clarity, structure, design, pertinence of pre-identified answers and length. The pre-test in the field also lead to better adapt some questions to the local dialect in order to facilitate understanding. The pre-tested standardized questionnaire was administered to the head or another representative of the household. Data collected were socio-economic characteristics, dog ownership and vaccination history, history of household member bite reported in the last 5 years preceding the study (characteristics of the bitten person, behaviors adopted after the bite, time to seek care, type of care, prevention methods that were adopted after the bite), knowledge and practices towards rabies, knowledge of the presence of an ARTC in the vicinity, its location and the type of treatment delivered. Questions were read out to the respondents in Malagasy by trained interviewers.

#### Knowledge score

A knowledge score was defined to rank the respondents’ answers to questions relative to rabies transmission, clinical signs in humans and animals and prevention which can impact practices, prevention and control and help design awareness campaigns. It comprised 10 questions (i) “Can animals transmit rabies?” (2 points), (ii) “Which animals can transmit rabies?” (1 point/right answer), (iii) “How can animals transmit rabies?” (1 point/right answer), (iv) “Can transmission occur through a non-bleeding wound due to a bite?” (1 point) or (v) “by ingesting cooked meat or milk from a rabid animal?” (2 points), (vi) “Is rabies fatal after the onset of clinical signs?” (2 points), (vii) “Can rabies be cured?” (2 points), (viii) “What are the clinical signs of rabid animals?” (1 point/right answer), (ix) “Is human vaccination efficient?” (1 point), (x) “Are traditional treatments efficient?” (1 point). No points were given to wrong or “do not know” answers and respondents who gave complementary correct answers were given 0.25 points per answer. The maximum score was 26 points (without counting complementary correct answers).

#### Practice score

A practice score was also defined to rank practices based on 3 questions (i) “What should a person bitten by a dog do?” (1 point/right answer), (ii) “What can be done to prevent animal rabies?” (1 point/right answer), (iii) “What should you do with a dog who has just bitten someone?” (1 point/right answer). No points were given to wrong or “do not know” answers and respondents who gave complementary correct answers were given 0.25 points per answer. The maximum score was 20 points (without counting complementary correct answers).

#### Statistical analyses

A principal component analysis was done to create a socio-economic score for the central more urban area and another one for the remote rural area based on socio-economic variables such as house owner or tenant, type of house, type of wall, number of bedrooms, availability of kitchen, shower and toilet, drinking water supply, energy supply type, ownership of a mobile phone and a bike. The socio-economic score was calculated using the factor scores from the first principal component as described by Vyas and Kumaranayake (2006) [64]. As a first step, a descriptive analysis (means, frequencies and standard deviations) of the variables related to socio-economic level (ownership of a mobile phone, television, bicycle, bike, car, access to water and electricity, type of house, number of bedrooms…) in the MHURAM initial census questionary [63] was carried out in order to inform decisions on which variables to include in the analysis and potentially identify data management issues (such as coding of variables and missing values). Qualitative categorical variables (such as the type of wall or the type of house) were recoded into binary variables so they could be included in the principal component analysis. Similar nominal variables with low frequencies were combined together and similar variables with relatively high frequencies were kept as separate variables. All binary variables created from categorical variables were included, including those that had low frequencies but were not similar enough to other variables to be combined. We excluded durable assets that were initially binary when they were owned by less than 2% of households. Variables with high number of missing values were also excluded.

Principal component analysis was run using the correlation matrix to ensure that all data had equal weight. The output was a table of factor scores for each variable included. Using the factor scores from the first principal component as weights for each variable, a dependent variable was constructed for each household and considered as the household’s socio-economic score. The socio-economic score was then reclassified into two categories using the median as the threshold within each area: high and low socio-economic status.

The knowledge and practice scores were reclassified into two categories using the median as the threshold for the bivariate and multiple logistic regression analysis. This method is used in similar international studies [33,41,43,51].

Variables associated with knowledge and practice were recoded as “correct” or “not correct”, the latter category combining “wrong” and “do not know” answers. Bivariate analyses using Student’s t-test or the non-parametric Mann-Whitney test for continuous variables and χ2 test or Fisher’s exact test for qualitative variables were carried out to compare household characteristics between central and remote areas and to identify variables associated to knowledge and practice scores. Individuals with missing values were omitted as missing data were less than 5%. Variables associated with knowledge and practice scores with *p* values < 0.25 were considered for multivariable logistic regression analyses. Multivariable logistic regressions were carried out using a backward and forward elimination based on the AIC (Akaike Information Criteria). Variables with *p* values < 0.05 were considered significant in the final models. Goodness-of-fit of the model was assessed by calculating the area under the Receiver Operating Characteristic curve (AUC ROC), an AUC of 0.5 suggesting that the model does not enable discrimination of individuals with high *versus* low knowledge and practice scores and an AUC of 1 suggesting a perfect discrimination [65].

Spatial autocorrelation of the knowledge and practice scores and of the residues of the knowledge and practice score models was assessed by calculating Moran’s global I index using ArcMap and ArcToolbox components of ArcGis software (version 10.6, ESRI, 2018).

Bite incidence was calculated at household level. A crude estimate of bite incidence at individual level was calculated under the hypotheses that (1) each bitten person was only bitten once, (2) neglecting any changes in the number of people in the household during the past five years except for the equal dispatching of births during the 5 years for children under five years old. Crude individual bite incidence was obtained by dividing the number of people bitten by the estimated number of person-years in the investigated households.

Statistical analyses were performed on the R studio software (version 1.1.456, R Core Team, 2019) [66,67].

### Qualitative approach

#### Study design and data collection

The survey was carried out in urban and rural communes of Moramanga district in December 2018 and consisted in 36 semi-structured interviews targeting individuals (21 female, 15 male) bitten by a dog or scratched by a cat and treated at the Moramanga ARTC in 2017 (n = 28), owners of biting dogs (n = 3), biomedical managers at ARTC (n = 3, two doctors and one nurse,), and local authorities (*Tangalamena* or village priests) at the community level (n = 2) (Table 1).

**Table 1:** Socio-demographic characteristics of participants of the qualitative survey and themes addressed.

Participant category	Number and sex of participants	Communes of origin of participants	Main themes addressed during the interview
Individuals bitten by a dog or scratched by a cat	10 women and 7 men	Moramanga City (n = 4), Andasibe (n = 4), Moramanga suburbaine (n = 3), Morarano (n = 2), Sabotsy Anjiro (n = 2), Anosibe An’ala (n = 1), Tsirinala (n = 1)	- History of the biting or scratching incident (where, when, how, by which type of dog), - Perception of dog bite and cat scratch, - Home care and/or medical care seeking after a bite, drivers of care pathway, - Knowledge and sources of knowledge about dog rabies, - Biting or rabid dog management, - Attitudes towards dog vaccination.
Parents of bitten children	9 women and 2 men	Moramanga City (n = 5), Morarano Gare (n = 2), Ambohibary (n = 1), Marovoay (n = 1), Tsirinala (n = 1), Vodiriana (n = 1)	
Owners of biting dogs	2 women and 1 man	Moramanga City (n = 1), Ambohibary (n = 1), Morarano Gare (n = 1)	- History of the biting event, testimonial of a bite, - Care for the bitten individual by the owners, - Biting or rabid dog management in their village, - Attitudes towards dog vaccination.
Biomedical managers	3 men	Not specified	- Knowledge on rabies treatment and vaccination following a bite or scratch, - Management of exposed patients, - Perception of patient’s healthcare seeking behavior.
Traditional authority	2 men	Ambohibary (n = 1) and Morarano Gare (n = 1)	- Knowledge and perception on dog rabies within the community, - Management of exposed and suspect cases within the village.

The 28 bitten or scratched individuals were selected out of the 679 individuals who sought PEP at the ARTC in 2017 on the basis of 4 criteria: at least one vaccination carried out at the ARTC, the existence of a telephone number and/or an exact address on the registration file, the availability of the person for an interview, and accessibility of the locality by car. These 28 individuals were either bite or scratch victims or parents of bitten or scratched children (Table 1). The Regional Direction of Public Health and ARTC caregivers facilitated the identification and localization of these participants. These 28 individuals resided in 8 communes located between 5 and 30 km from Moramanga city (n = 27) and in the commune of Anosibe an’Ala, 70 km from Moramanga (n = 1).

The themes addressed depended of the category of the participant (Table 1). All the questions were open questions such as “How is rabies perceived in your village?”, “What happened when you were bitten by a dog?”, “What do you think of dog vaccination?”. All interviews were carried out in Malagasy, recorded digitally with the approval of each participant. Interviews were then transcribed and translated into French. A thematic analysis of the Malagasy transcripts was carried out using analyses grids with the aim to highlight recurrences and discrepancies in the participants’ discourses.

## Results

### Quantitative approach on knowledge and practices

#### Demographic profile of the study population

In total, 334 households were included: 170 households located less than 5 km away from the ARTC (central area) and 164 households located more than 15 km away from the ARTC (remote area) (Fig 1). The mean age of the respondents was 40.8 years old and the sex ratio Male-to-Female was 0.7 (Table 2). Mean age was similar in remote and central areas. There were significantly more women in the central area (*p* = 0.04). There were more people living in the same household (4.7 *vs*. 3.9) and more children under five-year-old (10.9% *vs*. 8.3%) in the remote area (*p =* 0.01).

**Table 2:** Socio-demographic characteristics and dog ownership of respondents.

	Central area	Remote area	Total	*P* value^1^
***Characteristics of households***				
Number (Nb) of households (HH)	170	164	334	
Nb of people in investigated HH	669	774	1443	
Mean Nb of people per HH *Mean +/- SD*	3.94 +/- 1.74	4.72 +/- 2.23	4.32 +/- 2.03	**<0.001**
Mean percentage of children < 5 years old (yo) per HH *Mean % +/- SD*	8.33 +/- 12.99	10.85 +/- 13.79	9.57 +/- 13.43	**0.01**
Children under 5 yo in HH *(n (%)*				**0.02**
- Yes	56 (32.9)	75 (45.7)	131 (39.2)	
- No	114 (67.1)	89 (54.3)	203 (60.8)	
Socio-economic status *n (%)*				0.87^2^
- High	85 (50.0)	84 (51.2)	169 (50.6)	
- Low	84 (49.4)	80 (48.8)	164 (49.1)	
- Unknown	1 (0.6)	0 (0.0)	1 (0.3)	
***Characteristics of respondents***				
Age *Mean +/- SD*	40.65 +/- 16.16	40.9 +/- 14.79	40.79 +/- 15.47	0.86^2^
Age class (in years) *n (%)*				0.23^2^
- < 15	3 (1.8)	4 (2.4)	7 (2.1)	
- [15–25[	22 (12.9)	11 (6.7)	33 (9.9)	
- [25–40[	64 (37.6)	62 (37.8)	126 (37.7)	
- [40–60[	50 (29.4)	62 (37.8)	112 (33.5)	
- ≥ 60	28 (16.5)	23 (14.0)	51 (15.3)	
- Unknown	3 (1.8)	2 (1.2)	5 (1.5)	
Sex ratio *(Male/Female)*	0.56	0.88	0.70	0.06^2^
Sex *n (%)*				**0.04** ^2^
- Female	107 (62.9)	86 (52.4)	193 (57.8)	
- Male	60 (35.3)	76 (46.4)	136 (40.7)	
- Unknown	3 (1.8)	2 (1.2)	5 (1.5)	
***Dog ownership during the past 5 years***				
Nb of HH which owned dogs *n (%)*	46 (27.1)	64 (39.0)	110 (32.9)	**0.02**
Nb of dogs owned	81	86	167	
Nb of dogs per HH *Mean +/- SD*	0.48 +/- 0.97	0.52 +/- 0.84	0.50 +/- 0.91	0.63
Mean Nb of dogs per dog-owning household (DOHH) *Mean +/- SD*	1.76 +/- 1.12	1.34 +/- 0.84	1.51 +/- 0.98	**0.04**
Nb of HH owning at least one vaccinated dog *n (% among DOHH)*	19 (41.3)	3 (4.7)	22 (20.0)	**<0.0001**
Nb of vaccinated dogs *n (%)*	36 (44.4)	3 (3.5)	39 (23.4)	**<0.0001**
Mean Nb of vaccinated dogs within DOHH *Mean +/- SD*	0.78 +/- 1.28	0.047 +/- 0.21	0.35 +/- 0.91	**<0.001**
***Current dog ownership***				
HH owning dogs *n (%)*	22 (12.9)	30 (18.3)	52 (15.6)	0.18
Nb dogs owned	40	42	82	
Mean Nb of dogs currently owned per HH *Mean +/- SD*	0.24 +/- 0.71	0.26 +/- 0.80	0.25 +/- 0.75	0.80
Mean Nb of dogs currently owned per DOHH *Mean +/- SD*	1.82 +/- 1.01	1.40 +/- 1.38	1.58 +/- 1.24	0.21
Age category of dogs *n (%)*				0.09
- Puppies < 3 months old (mo)	2 (5.0)	8 (19.0)	10 (12.2)	
- Dogs ≥ 3 mo	38 (95.0)	34 (81.0)	72 (87.8)	
Sex of dogs > 3 mo *n (%)*				0.14
- Female	20 (50.0)	12 (28.6)	32 (39.0)	
- Male	18 (45.0)	22 (52.4)	40 (48.8)	
HH owning at least one vaccinated dog *n (% within DOHH)*	15 (68.2)	1(3.3)	16 (30.8)	**<0.0001**
Vaccinated dogs *n (%)*	25 (62.5)	1 (2.4)	26 (31.7)	**<0.0001**
Mean Nb of vaccinated dogs within DOHH *Mean +/- SD*	1.14 +/- 1.21	0.03 +/- 0.18	0.50 +/- 0.96	**<0.001**
Overall dog-to-human ratio	0.060 (1:16.7)	0.054 (1:18.4)	0.057 (1:17.6)	
Mean dog-to-human ratio per HH (95% CI)	0.063	0.057	0.060	0.79
	(0.032–0.093)	(0.027–0.087)	(0.038–0.081)	
***Awareness***				
Prior awareness of rabies *n (%)*	157 (92.4)	150 (91.5)	307 (91.9)	0.77
Benefited from rabies awareness campaign *n (%)*	51 (30.0)	19 (11.6)	70 (21.0)	**<0.0001**
Aware of the existence of the ARTC *n (%)*	31 (18.2)	18 (11.0)	49 (14.7)	0.06

^1^*P* values ≤ 0.05 are presented in bold.

^2^*P* values were calculated without taking into account individuals with missing values

Out of the 24 variables initially collected to describe the socio-economic status, 19 and 12 were kept to define the socioeconomic status in the central and remote areas respectively because either less than 2% of households indicated owning these assets (such as a landline or a truck for example) or because there were too many missing values (one variable, owning a generator). The variables retained for each area, the values for each variable of the two first components and the loadings of the principal component analysis are presented in S1 Table. For one individual, the socio-economic score could not be calculated because the values of the socio-economic variables were all missing.

As the calculation of the socio-economic status differed between each of the two areas, no significant difference of distribution between high and low socio-economic statuses were expected (*p =* 0.87).

#### Dog ownership

On average, at the time of the study, 15.6% of households owned dogs and these households owned an average of 1.6 dogs (Table 2). Considering only owned dogs, the overall dog-to-human ratio was 0.057 (*i*.*e*., one dog for 17.6 humans). Difference between mean dog-to-human ratios per household in the remote area and central area was small and not statistically significant. There were more dog owning households in the past five years in the remote area (39.0 *vs*. 27.1%, *p =* 0.02). The percentage of vaccinated dogs was 26-fold greater in the central area *vs*. the remote area (62.5% *vs*. 2.4%) and this difference had increased compared to the situation 5 years before when it was 13-fold greater (44.4% *vs*. 3.5%). The detailed reasons for not vaccinating dogs are presented in S2 Table. Among the 27 households of the remote area which owned only unvaccinated dogs, the main reasons not to vaccinate their dogs were the distance to a veterinarian, cited by 11 respondents (41%), followed by not knowing it was possible to vaccinate dogs (four respondents, 15%), the lack of funds to pay for the vaccine (three respondents, 11%) and the fact, according to respondents, that it is uncommon to vaccinate dogs in rural areas (three respondents, 11%). In the 7 households of the central area which owned only unvaccinated dogs, none mentioned distance as a barrier to vaccination. Reasons for not vaccinating were diverse in the central area (cost, time constraint, didn’t know it was possible to vaccinate dogs or didn’t think it was useful) (S2 Table). Most of the respondents (n = 305, 91.3%, *p =* 0.06 between the two areas) believed dog vaccination could prevent rabies transmission. One respondent (0.3%) answered that the vaccination could give rabies to the dog and another said that if they vaccinated their dog, thieves would no longer be afraid of the dog. Significantly more people had benefited from a rabies awareness campaign in the central area (30.0% *vs*. 11.6%, *p*<0.0001). Most respondents had prior awareness of rabies (91.9% in both central and remote areas) but only 14.7% were aware of the existence of the ARTC. The difference between the percentages of people who were aware of the existence of the ARTC was not statistically significant but close to the 0.05 threshold (*p =* 0.06) between the remote (11.0) and the central areas (18.2).

#### Knowledge score

Table 3 presents the answers to questions relative to the knowledge score. The following *p* values mentioned hereafter compare the percentage of respondents giving correct answers in the remote and central areas (see S3 Table for detailed distribution in the two areas). The majority of respondents (93.7% (313/334)) knew that animals could transmit rabies (96.7% (297/307) among the respondents who had prior awareness of rabies) (*p =* 0.035, 96.5% in central *vs*. 90.9% in remote areas). Among the 313 respondents who knew animals could transmit rabies, 98.7% knew that dogs could transmit rabies (*p =* 0.6), 75.7% knew that cats could transmit rabies but only 29.4% knew that ruminants could also transmit rabies. Although more respondents from the remote area knew that ruminants could transmit rabies (*p =* 0.002), this percentage remained low in both areas (37.6% *vs*. 22.0%). Significantly more respondents from the central area knew that lemurs could transmit rabies (26.2% *vs*. 9.4%, *p*<0.001) and that rodents didn’t (45.7% *vs*. 28.2%). Respondents knew that rabies could be transmitted through bites (97.9%), scratches (80.0%) or by a lick on a wound (70.9%, with significantly (*p =* 0.006) more respondents in the central area) but only 21.8% knew that it could be transmitted by manipulating meat and 83.5% believed that eating cooked meat or milk from a rabid animal was at risk. Most respondents cited aggressive behavior (86.2%, with more participants from the remote area citing this symptom, 92.1% *vs*. 80.0%, *p =* 0.002) and drool (88.0%) as clinical signs of rabies in animals. Most respondents (95.5%) knew that rabies was fatal, however, to the question if rabies was curable, the majority also answered yes (90.4%). Most of the respondents believed that the human rabies vaccine was effective and only 4.5% thought that traditional treatments were effective.

**Table 3:** Knowledge score details.

	Score for correct answer	Answers: N (%)			Respondents *n*	*P* value^a^ Central/ Remote
		No	Yes	Do not know		
Can animals transmit rabies?	2	2 (0.6)	313 (93.7)^b^	19 (5.7)	334	**0.04 C**
If yes, which animals?					313	
Ruminants	1	115 (36.7)	92 (29.4)	106 (33.9)		**0.002 R**
Dogs	1	1 (0.3)	309 (98.7)	3(1.0)		0.62
Cats	1	33 (10.5)	237 (75.7)	43 (13.8)		0.20
Lemurs	1	95 (30.4)	57 (18.2)	161 (51.4)		**<0.001 C**
Birds/wild birds	1	152 (48.6)	14 (4.5)	147 (46.9)		0.10
Rodents	1	117 (37.4)	75 (24.0)	121 (38.6)		**0.001 C**
*Extra points* ^c^						
*Bat*	*0*.*25*		*1 (0*.*3)*			
*Pig*	*0*.*25*		*1 (0*.*3)*			
*Fossa (Cryptoprocta ferox)*	*0*.*25*		*1 (0*.*3)*			
How is rabies transmitted?					334	
Touching sick animals	1	262 (78.4)	39 (11.7)	33 (9.9)		0.86
Bite	1	1 (0.3)	327 (97.9)	6 (1.8)		0.28
Scratch	1	37 (11.1)	267 (80.0)	30 (8.9)		0.05
Meat manipulation	1	193 (57.8)	73 (21.8)	68 (20.4)		0.07
Animal licking a wound	1	49 (14.7)	237 (70.9)	48 (14.4)		**0.006 C**
Contact with urine/feces	1	213 (63.8)	44 (13.2)	77 (23.0)		0.89
Can rabies be transmitted through a non-bleeding wound caused by a dog bite?	1	53 (15.9)	242 (72.4)	39 (11.7)	334	0.84
Can rabies be transmitted by ingesting cooked meat or milk from a rabid animal?	2	18 (5.4)	279 (83.5)	37 (11.1)	334	0.06
What are the clinical signs of animal rabies?					334	
Underweight	1	233 (69.8)	70 (21.0)	31 (9.3)		0.20
Aggressive		39 (11.7)	287 (85.9)	8 (2.4)		**0.002 R**
Drool		16 (4.8)	294 (88.0)	24 (7.2)		0.43
*Extra points* ^c^						
*Confusion*^d^	*0*.*25*		*44 (13*.*2)*			
*Unusual vocalisation*	*0*.*25*		*11(3*.*3)*			
*Paralysis*	*0*.*25*		*8(2*.*4)*			
*Agitation*, *hyperactivity*	*0*,*25*		*5(1*.*5)*			
*Unusual/fixed gaze*	*0*.*25*		*3(0*.*9)*			
Is rabies fatal?	2	5 (1.5)	319 (95.5)	10 (3.0)	334	0.74
Is rabies curable?	2	10 (3.0)	302 (90.4)	22 (6.6)	334	0.10
Is human vaccination effective against rabies?	1	46 (13.8)	244 (73.1)	44 (13.1)	334	0.24
Is traditional medicine effective against rabies?	1	246 (73.7)	15 (4.5)	73 (21.8)	334	0.66

^a^ The *p* value compares the variables between remote and central areas (*p* values < 0.05 are in bold). C (central) and R (remote) indicate the area in which more correct answers were observed.

^b^ Correct answers are represented with shaded areas.

^c^ 0.25 extra points were given for other correct answers given.

^d^ Confusion included "disorientation, runs without direction, does not recognize its owner or commands, behaviour change, distraught"

The median score for correct responses regarding rabies knowledge was 16.25 (min = 0, [Q1 = 15; Q3 = 18,] max = 22) and the average score was 15.8 (+/-SD = 3.4). The central area had a better (*p*<0.002) knowledge (mean = 16.41 (+/-SD = 2.73)) than the remote area (mean = 15.21 (+/-SD = 3.82)).

Results of bivariate analyses showing which variables were associated to the knowledge score are displayed in S4 Table. The variables socio-economic status (*p =* 0.001), type of area (*p =* <0.01), having prior awareness of rabies (*p =* 0.07), being aware of the existence of the ARTC (*p* = 0.10), having benefited from rabies awareness campaign (*p =* 0.12) and having owned a dog in the previous 5 years (*p* = 0.19) were considered for the multivariable logistic regression analyses. The knowledge score on rabies of current dog owners and respondents from households which did not own a dog was not statistically significantly different.

Table 4 shows the results of the final multivariable logistic regression model for the knowledge score on rabies. The model with the lowest AIC included four variables: having a higher socio-economic status, living in the central area, having prior awareness of rabies and having owned a dog during the last 5 years were associated with a higher knowledge score (although the associations of the last two covariates with the knowledge score were not significant). Detailed model selection is presented in S5 Table. Alternative model obtained in the upward stepwise approach was very similar as it included the three first covariates. The area under the ROC curve of the final model was 0.65 (95% CI: 0.59–0.70). Although spatial autocorrelation analyses based on Moran’s I global index showed that the knowledge score displayed significant spatial clustering (*p* = 0.004) when assessed for the entire zone, no significant spatial autocorrelation was detected for the knowledge score within the central and remote areas individually (S6A Table). Importantly, no significant spatial autocorrelation of the residues of the knowledge score model was detected at the three scales tested (entire, central and remote areas) (S6B Table).

**Table 4:** Multivariable logistic regression model for the rabies knowledge score.

	Respondents n (%) with a knowledge score < / ≥ median (N = 333*)	*P* value	Adjusted OR (95% CI)
**Socio-economic status**		**0.001**	
- Low	96 (58.18) / 68 (40.48)		1
- High	69 (41.82) / 100 (59.52)		**2.08** (1.33–3.26)
**Area**		**0.005**	
- Remote	93 (56.36) / 71 (42.26)		1
- Central	72 (43.64) / 97 (57.74)		**1.91** (1.22–3.00)
**Prior awareness of rabies**		**0.16**	
- No	18 (10.91) / 9 (5.36)		1
- Yes	147 (89.09) / 159 (94.64)		**1.85** (0.81–4.49)
**Having owned a dog during the last 5 years**		**0.11**	
- Remote	116 (70.30) / 107 (63.69)		1
- Central	49 (29.70) / 61 (36.31)		**1.49** (0.92–2.42)

* Analysis was performed on 333 individuals as one individual with no value for the socio-economic status was removed.

#### Practice score

Overall, 142 respondents (42.5%) knew someone who had been bitten. Yet, only 25 (7.5%) households (consisting of 17 households from the central area (10.0% of households of the central area) and 8 households of the remote area (4.9% of households of the remote area)) out of 334 answered that a member of the household had been bitten by an animal in the last 5 years (the difference is not statistically significant at the household level, *p* = 0.08). In a further 6 (central) households, someone visiting the household had been bitten. Bites concerned only one household member each time. Bite event numbers within the households (n = 17 in central and 8 in remote area) were divided by the person-years in the households (3213 and 3686 person-years in central and remote areas respectively), giving a crude estimated bite incidence of 0.53 per 100 person-years (95% CI: 0.31–0.85) in the central area and of 0.22 per 100 person-years (95% CI: 0.09–0.43) in the remote area (*p =* 0.03).

Bite victim characteristics and practices are presented in S7 Table. The mean age of the 31 bitten people was 22 years old (+/-SD = 17, range 1–50). Out of them, 4 were children under the age of 5 (13%). All 8 bite events in the remote area occurred outside the house, as well as 15/23 (65%) of bite events in the central area. Most attacks were not provoked (n = 19, 61%). Twenty respondents (65%) answered that the person consulted a health care center after the bite, 16 (52%) benefited from PEP (4/6 visitors in the central area, 7/17 households of the central area and 5/8 households of the remote area) and the average time to seek a health care center was 0.6 days (+/-SD = 0.82). Reasons for not consulting a health care center (11 people) were the cost (n = 2), the wound was small (n = 2), the victim could not leave his/her work or occupation (n = 2), the distance to the health care center (n = 1, from the remote area), the victim was a visitor of a household in which one member was a doctor (n = 1), the dog was thought to be too young to be at risk (n = 1) or was vaccinated against rabies (n = 1) or it was brought to be treated by a traditional practitioner after the bite (n = 1) (see S7 Table). The average number of times the person had to return to the health care center to get PEP was 3.6 times (+/-SD = 0.81). Four persons (13%) had a complication after the bite. Among them 3 had not visited a health center after the bite. Thirty of the 31 (97%) bite victims were healthy 3 months after the bite and one (3%) was deceased. The deceased person was a visitor who had not gone to a health center after being bitten because someone in the visited household was a doctor. We do not have more information on the cause of death.

Table 5 shows the details of the practice score for the 334 respondents (see S8 Table for detailed distribution in the two areas). The median score for correct responses towards practices to prevent rabies was 12 (min = 4, [Q1 = 11; Q3 = 13], max = 16) and the average score was 11.8 (+/-SD = 1.78). Practice score was significantly lower (*p*<0.001) in the remote (mean = 11.2 (+/-SD = 1.52)) compared to the central area (mean = 12.3 (+/-SD = 1.84)).

**Table 5:** Practice score details.

	Score for correct answer	Answers (*n* = 334)			*P* value^a^ Central/ Remote
		No	Yes	Do not know	
What should a bitten person do					
Nothing	1	324 (97.0)^b^	7 (2.1)	3 (0.9)	0.75
Wash the wound	1	176 (52.7)	152 (45.5)	6 (1.8)	**0.01 C**
Consult a traditional healer	1	328 (98.2)	1 (0.3)	5 (1.5)	>0.99
Apply mud on the wound	1	329 (98.5)	0 (0.0)	5 (1.5)	0.68
Apply cooked rice on the wound	1	325 (97.3)	2 (0.6)	7 (2.1)	0.33
Call or consult a doctor	1	234 (70.0)	97 (29.1)	3 (0.9)	0.12
Call or consult a veterinarian	1	316 (94.6)	15 (4.5)	3 (0.9)	**0.02 C**
Seek a medical center	1	132 (39.5)	199 (59.6)	3 (0.9)	**0.02 C**
Seek PEP*	1	308 (92.2)	22 (6.6)	4 (1.2)	**<0.001 C**
Isolate dog to place it under observation	1	311 (93.1)	19 (5.7)	4 (1.2)	0.88
Screen the dog for rabies	1	306 (91.6)	22 (6.6)	6 (1.8)	**0.008 R**
Kill the dog	1	247 (73.9)	83 (24.9)	4 (1.2)	0.41
*Extra points* ^c^					
*Advise the victim to seek for care*	*0*.*25*		*1 (0*.*3)*		
*Advise the dog owner to isolate the dog*	*0*.*25*		*1 (0*.*3)*		
How can we prevent animal rabies					
Vaccination	1	15 (4.5)	305 (91.3)	14 (4.2)	0.14
Feeding the animal	1	253 (75.6)	53 (15.9)	28 (8.5)	0.84
*Extra points*					
*Bring it to the veterinarian*	*0*.*25*		*1 (0*.*3)*		
What should you do if a dog you know has bitten someone					
Consult or call a veterinarian*	1	269 (80.5)	64 (19.2)	1 (0.3)	**<0.001 C**
Consult or call a doctor	1	242 (72.5)	92 (27.5)	0 (0.0)	**<0.001 C**
Consult a traditional healer	1	334 (100)	0 (0.0)	0 (0.0)	/
Isolate the dog	1	299 (89.5)	35 (10.5)	0 (0.0)	0.67
Kill the dog*	1	170 (50.9)	163 (48.8)	1 (0.3)	**<0.001 C**
Nothing	1	310 (92.8)	23 (6.9)	1 (0.3)	0.35
*Extra points*					
*Wash the wound*	*0*.*25*		*1 (0*.*3)*		
*Send the victim to Institut Pasteur*	*0*.*25*		*1 (0*.*3)*		
*Advise the owner to consult a veterinarian*	*0*.*25*		*5 (1*.*5)*		

^a^ The *p* value compares the variables between remote and central areas (*p* values < 0.05 are in bold). C (central) and R (remote) indicate the area in which more correct answers were observed.

^b^ Correct answers are represented with shaded areas.

^c^ 0.25 extra points were given for other correct answers given.

Following a dog bite, only 45.5% of the respondents said they would wash the wound with significantly more people washing the wound in the central area (*p =* 0.011). Overall, 87.7% of people would seek care from conventional medicine: around 30% would seek medical advice from a doctor, nearly 60% would go to a medical center and 6.6% would seek PEP. Intention to seek care was significantly higher in the central area towards PEP (11.8% *vs*. 1.2%, *p*<0.001), medical centers (66% *vs*. 53%, *p =* 0.017) and veterinarians (7.1 *vs*. 1.8%, *p =* 0.021). S9 Table compares the practices carried out by the 25 members of households who had been bitten to what the respondents said a bitten person should do after a bite. Overall, only 45 respondents (13.5%) mentioned at least one unconventional method to treat the wound (the most frequently cited method being applying oil (38 respondents). Opposite, human rabies vaccination was considered efficient by 73.1% of respondents and 305 (91.3%) said that dog vaccination prevented animal rabies. Yet only 5.7% of respondents would isolate the dog, 4.5% would consult a veterinarian and 24.9% would kill the dog.

Concerning practices to adopt if a known dog bit a person, 163 respondents (48.8%) answered they would kill the dog (with significantly less respondents saying they would kill the dog in central areas, 64% *vs*. 37%, *p*<0.01) and only 64 respondents (19.2%) would call a veterinarian (with a significantly higher rate in the central area (29% *vs*. 9.1%, *p*<0.001)). Significantly higher rates of respondents would call or consult a doctor in the central area (40% *vs*. 15%, *p*<0.001).

Results of bivariate analyses showing which variables were associated with the practice score are shown in S10 Table. The variables gender (*p =* 0.09), area (*p*<0.001), socio-economic status (*p =* 0.06), dog ownership in the last five years (*p =* 0.12), having prior awareness of rabies (*p =* 0.02), being aware of the existence of the ARTC (*p =* 0.002) and having benefited from a rabies awareness campaign (*p =* 0.02) were considered for the multivariable logistic regression analyses as explanatory variables of a greater practice score (practice score > = median). Opposite, respondents who currently owned dogs did not have better practices than respondents who did not own dogs (*p =* 0.9). Respondents with a higher knowledge score also had a significantly higher practice score (*p =* 0.002).

Table 6 shows the final multivariable logistic regression model for the practice score (detailed model selection process is presented in S11 Table). Living in the central area, having prior awareness of the ARTC’s existence and of rabies, and having a high socio-economic status were associated with a higher practice score (although the associations of the last two covariates with the practice score were not significant). Alternative model obtained in the upward stepwise approach was very similar as it included the three first covariates. The model had an acceptable discrimination capacity (AUC ROC = 0.73; 95% CI: 0.68–0.79). Although the practice score displayed significant spatial clustering (*p*<10^−6^) when assessed for the entire zone, no significant spatial autocorrelation was detected for the knowledge score within the central and remote areas individually (S6C Table). Importantly, no significant spatial autocorrelation of the residues of the practice score model was detected at the three scales tested (entire, central and remote areas) (S6D Table).

**Table 6:** Multivariable logistic regression model for the practice score.

	Respondents n (%) with a practice score < / ≥ median (N = 328*)	*P* value	Adjusted OR (95% CI)
**Area**		**<0.0001**	
- Remote	111 (68.10) / 51 (30.91)		1
- Central	52 (31.90) / 114 (69.09)		**4.78** (2.98–7.77)
**Awareness of the ARTC’s existence**		**0.02**	
- No	149 (91.41) / 131 (79.39)		1
- Yes	14 (8.59) / 34 (20.61)		**2.29** (1.14–4.80)
**Prior awareness of rabies**		**0.09**	
- No	18 (11.04) / 8 (4.85)		1
- Yes	145 (88.96) / 157 (95.15)		**2.21** (0.90–5.85)
**Socio-economic status**		**0.12**	
- Low	88 (53.99) / 73 (44.24)		1
- High	75 (46.01) / 92 (55.76)		**1.47** (0.91–2.38)

* Analysis was performed on 328 individuals as one individual with no value for the socio-economic status and five individuals with no values for the sex were removed.

### Qualitative survey

#### Types of human-dog interaction

The results of the qualitative study highlight the existence of five categories of dogs, which are differentiated by their interactions and proximity with humans, whether they are owned or not, whether they are free to roam or not, their role and where they are present (central and/or rural areas) (Table 7). The first category consists in unowned free-roaming dogs, present in both rural and central areas and perceived as representing a threat or at least a nuisance to humans. It includes feral dogs (*i*.*e*., domestic dogs which have reverted to the wild state and are no longer directly dependent upon humans). The four other categories are all owned dogs. The second category consists of free-roaming guard dogs, present in rural and, to a lesser extent, in urban areas. They belong to an individual owner or to a community with which they are familiar. Yet, in the opinion of interviewees, as all free-roaming dogs, they represent a threat, even if they live relatively close to humans. The third category consists of restrained guard dogs. They are confined on the owner’s property (often in the courtyard) and they have no contact with humans or only at a distance. The fourth category consists in free-roaming hunting dogs, present exclusively in the remote areas. The majority of dogs fall into the two categories of guard dogs as for most families the primary function of a dog is to protect the household and belongings against the intrusion of strangers. The last category is exclusively encountered in urban areas and consists in pet dogs which are considered as members of the family and cared for, the owners being responsible of their well-being, feeding, grooming and medical care including vaccination. Very few dogs belong to this category.

**Table 7:** Dog typology.

Category	Owned/ unowned	Dog mobility	Role	Location	Proximity to humans
1	Unowned	Free-roaming	/	Rural and urban	Very low to null
2	Owned (individual or community)	Free-roaming	Guard dog	Rural (and urban but lesser extent)	Familiar
3	Owned	Restrained	Guard dog	Urban	Familiar but low contacts
4	Owned	Free-roaming	Hunting dog	Rural	Familiar
5	Owned	Restrained	Pet dog	Urban exclusively	Very close proximity

Despite half of the bite victims, parents of bitten children and owners of biting dogs (n = 10/21) who discussed this topic mentioned dog vaccination as a means to prevent rabies transmission, the majority of dogs are not vaccinated. The main reason for not vaccinating a dog against rabies is that the dog is considered harmless by its owners and “does not bite”. The second reason for not vaccinating is the cost: budget for dog vaccination is not a priority in the household’s budget. As a 60-year-old man said: “today’s work will allow us to buy food for the day. How can we find money for dog’s shots?”. Dog vaccination is considered as something intended for rich families who live in close contact with their dog. Three people in the remote rural area replied “it is not in our habits in rural areas”. Qualitative interviews suggest that the main perceived advantage of rabies vaccination is avoiding the social disruption and financial impacts in the event of a bite. Indeed, if a dog bites someone, social pressure will be strong on the owner to incite him/her to take in charge health costs after exposure.

#### Practices concerning bite management: Care pathway

Of the 28 people bitten, 19 were bitten by free-roaming dogs, 7 by guard dogs and 2 by pet dogs. No bites were made by feral dogs even though this category represented the greatest threat in terms of bites in the eyes of the interviewees.

Out of the 28 bite victims, rabies was confirmed in the biting dog for 7 of them. Among the 7 victims of a confirmed rabid dog, three were bitten by someone else’s dog and four by their own dog. The three bitten by someone else’s rabid dog went to the nearest local health center (a basic health care center level II, called “centre de santé de base II” or “CSBII” in Madagascar) before being referred to the ARTC in Moramanga hospital. Among the four who had been bitten by their own rabid dog, one went to a CSBII before being referred to the ARTC and three went directly to the ARTC, on the day following the bite or a few days later in one case. In 5 out of 7 cases, the dog was suspected of rabies because it had already bitten 2 or 3 other people the same day. In the 2 remaining cases, the dog presented an unusual behavior, exhibiting signs of restlessness or aggressiveness (biting without reason unusual objects such as dry banana leaves), drool and “mad eyes”. Rabies was confirmed only after the bite victims had gone to the ARTC for vaccination, by post-mortem laboratory analyses on brain samples. The dogs had either been slaughtered or died of the disease a few days after having bitten people. Some had been buried and were dug-up for the post-mortem analyses. If the dog was declared vaccinated by its owner, which was very rare, the bite was not considered as being at risk and no biomedical care was sought.

Most respondents said that wounds caused by a dog bite should be washed with soap and almost all of them declared doing so to get rid of “microbes” or the “poison” injected by the dog’s teeth. If the bitten person considered that the dog was not rabid (and the bite was “accidental”) or because of unawareness, washing with soap and putting hair styling oil, kitchen oil or zebu fat on the wound was considered sufficient. Respondents considered that superficial bites due to known or unknown dogs could be ignored and left unwashed. Most respondents (15/28) considered that bitten people should immediately seek the dog’s owner to know if the dog was vaccinated, to ask him to treat the wound (wash it with soap and apply oil) and to support treatment costs. On the contrary, if the dog was a free-roaming dog or a feral dog, the victim should be brought to a health center, either after or without washing the wound with soap.

For the biting dog, in most cases, the biting event had no consequences. Contrary to common belief, dogs were rarely killed after a single bite. Out of the 5 respondents who said they had killed the biting dog, four of them were owners who had been bitten by their own dog. Surveillance of biting dogs through confinement and daily observation for clinical signs of rabies were very rarely mentioned or carried out by respondents. Only 2/28 tied the dog to monitor it as recommended by the vet.

Three factors influenced medical care seeking behavior after a bite event. The first factor was the advice given by acquaintances, the community or the family. Most patients said they had followed the advice to go to a health center. Some acquaintances had mentioned rabies risk. Advice coming from family members was particularly valued. If a rabies case had occurred within the community, the story of that event encouraged the bite victim to seek medical care. Secondly, if the dog owner accepted to cover costs (8/28), the bite victim went to the health center. Finally, the last factor which influenced the care pathway was whether the dog was perceived as rabid (drool on the dog’s muzzle, mad eyes), in which case the victim, eventually under the pressure of his/her family, went to the health center more rapidly than otherwise.

## Discussion

The aim of this study was to assess KAP towards rabies in Moramanga district. In particular, the quantitative approach aimed to assess if distance to an ARTC, among other factors, impacted knowledge and practices, and using a complementary qualitative approach, we aimed to better understand (i) attitudes toward dogs, dog bites and dog vaccination, (ii) care seeking behavior after a bite and (iii) biting dog management practices. KAP surveys can provide valuable descriptive information on complex issues and can help assess what aspects of a problem can be improved through awareness campaigns. Quantitative KAP studies are sometimes criticized because they fail to explain the logic behind people’s attitudes and practices. Opposite, qualitative studies provide more detailed information through in-depth interviews with key informants which can help better understand the logic behind some behaviors. Yet, they are sometimes criticized because the number of people interviewed is usually small and not randomly sampled and therefore not considered as representative of a wider population. This is illustrated in this qualitative study, as the bite victims interviewed were recruited through the ARTC and they are thus not representative of all bite victims of Moramanga, in particular they are not representative of those who did not seek care. These qualitative approaches are not designed to produce numerical estimates and statistical models but they can be useful to understand the reasons underlying attitudes and behaviors. To better address the limitations inherent to each type of KAP surveys, we combined complementary quantitative and qualitative approaches on the topic so that the latter could shed light on the context and clarify answers to the quantitative questionnaire, as in Sikana *et al*. (2021) [45].

### Dogs: Population estimates, types and vaccination

Dog populations have seldom been studied in Madagascar yet results on dog ownership show considerable heterogeneity. A study carried out in the capital city in 2007 showed that dog ownership was very common (88.9% of households) and the dog-to-human ratio was 1:4.5 [31]. The previous study carried out in Moramanga city in 2011 had found that 23.2% of households owned at least one dog and that the mean dog-to-human ratio was 1:7.2 [8]. Our results in the central area, in or very close to the urban commune of Moramanga, show an even smaller percentage of dog-owning households (12.9%) and a lower dog-to-human ratio (1:16.7). It is difficult to draw conclusions on eventual trends as these estimates were obtained in two areas at two different time points. They might indicate that (i) there could be less dogs per habitant in the provincial city of Moramanga than in the capital city, and/or that (ii) the number of dogs per habitant could have strongly declined over the years (- 51.1% compared to 2011) in Moramanga. To assess these two hypotheses, it would be necessary to determine whether a similar decline in the number of dogs per habitant was also observed in Antananarivo since 2007 and to collect more data on dog population estimates from different areas in Madagascar. Apart from focusing in only one district, another limit of this study is that we did not assess the relative importance unowned dogs *versus* owned ones. Future studies should therefore include several districts and include the estimation of unowned dogs. In a context where most dogs are free-roaming, it is often difficult to be certain that a dog has no owner, yet this information is very useful to plan dog vaccination campaigns.

Within Moramanga district, differences in dog-to-human ratios between remote and central areas were not statistically significant, perhaps because we were comparing (i) a central area which consisted in a provincial town and not a very dense urban area and (ii) a remote area within the district, where households were less than 30 km away from the ARTC. In Africa, dog-to-human ratios are often higher in rural areas than in urban areas [68–71]. Moramanga dog-to-human ratio was closer to values usually observed in urban African areas as in Tanzania [70], Kenya [72] or Chad [73]. To assess whether differences between urban and rural areas are less important in Madagascar than elsewhere in Africa, more data on dog populations in Madagascar need to be collected.

The study of Rakotonirainy showed that the dog-to-human ratio displayed strong heterogeneity within Moramanga city: it ranged from 1:4.2 to 1:19 depending on the fokontany (infra-communal division) [8]. Factors driving these differences were not explored. Paucity of data on estimates of dog population in Madagascar and heterogeneity of estimates [8,74] both call for more studies on this topic to better inform dog vaccination campaigns. Precise estimates of dog populations, dog demographic parameters such as lifespan, as estimated in Cambodia [74], mapping of dog populations at the national scale, as done in Thailand [75] would be particularly useful to parametrize rabies transmission models and identify most efficient vaccination strategies and resource allocation.

Five categories of dogs were identified in Moramanga: free-roaming unowned dogs, two categories of guard dogs (free-roaming and restrained), free-roaming hunting dogs and the less common pet dogs. As in Valenta *et al*. (2016) and Rakotonirainy (2012), the main role of dogs was personal and property protection [8,24]. Unsurprisingly, attachment to dogs was low [11] and, free-roaming unknown dogs were considered more at risk of contracting rabies than owned well-known dogs [11,76], although data collected in this study and in other studies carried out in Madagascar [8,10] suggest that most bite incidents are due to owned dogs.

The qualitative survey showed that most dogs were not vaccinated, as in most sub-Saharan African settings [1]. Yet the quantitative study showed that a surprisingly high percentage of dogs were declared as vaccinated in the central area (62.5% *vs*. 2.4%). This high vaccination coverage in the central area could have resulted from the 2018 vaccination campaign (over 1,700 dogs vaccinated) carried out in Moramanga city by the non-governmental organization Mad Dog Initiative with Madagascar’s Ministry of Public Health, Ministry of Agriculture, Livestock and Fisheries and other collaborators [32]. Because of this campaign, vaccination coverage in Moramanga city commune should not be considered as representative of other urban areas of the country. In 2011, Rakotonirainy had found that 37% of the dogs in Moramanga city (urban commune) were declared vaccinated by their owner and 11.7% had a vaccination card proving that the dog was vaccinated [8]. These estimates are more in line with results obtained in Antananarivo in 2014–2015 where 25% of the dogs were declared vaccinated [58]. In rural areas, where 80% of the population lives, a coverage under 5% was expected, in line with the low coverage estimated by Hampson *et al*. (2015) for the country [1]. In any case, even if the vaccination coverage in Moramanga city is higher than in the outlying villages, it remains under the 70% coverage threshold needed to control and eventually eliminate the disease [3,77] and the fact that the city is surrounded by areas where the coverage is extremely low jeopardizes control efforts in the long term. Mass dog vaccination is the keystone of rabies control and elimination [3], and campaigns should be repeated regularly enough to ensure that herd immunity is maintained despite dog population turnover [3]. They should also take into account the local ecology of dogs, including whether they are confined or free-roaming, owned by individuals, communities or unowned. Mass dog vaccination campaigns are of utmost importance as dog-mediated rabies can’t be eliminated without them. They have repeatedly been shown to be effective, opposite to dog culling which does not reduce dog density or rabies circulation in the long term [3].

The qualitative survey confirmed that dog vaccination was not a priority because of the weak relations between humans and dogs within the household and of the vaccination cost. In the quantitative study, the main barriers to vaccination were the distance to a place where dogs could be vaccinated (especially in the remote rural area), followed by the cost of vaccination. Results on the distance barrier are in line with Beyene *et al*. (2018) who showed that distance from veterinarians was the best predictor for the intention to vaccinate animals [34]. They are also in line with Castillo-Neyra *et al*. (2017) who identified distance to the vaccination point and difficult topography in peri-urban areas of Arequipa, Peru as barriers to vaccination [78]. They are also in line with recent work in Moramanga district which compared two vaccination deployment strategies and showed that vaccine provided by the veterinarian during routine visits in the village achieved better spatial coverage than static vaccination points [32] and with a study carried out in Menabe (western part of Madagascar) where physical accessibility to the ARTC and to canine vaccines at the district level were identified by the interviewees as major levers to improve rabies prevention [79]. In Madagascar, the cost of vaccine was also showed to be an important barrier by some authors [58,79].

The identification of cost and access to animal health practitioners as important barriers to dog vaccination suggests that awareness raising campaigns promoting dog vaccination will not succeed on their own. Indeed, the most effective way to overcome cost and access issues is free-of-charge mass dog vaccination designed to maximise spatial outreach. Filla *et al*. (2021) showed that (i) past vaccination campaigns in Madagascar were well accepted by communities if the vaccination was free but fewer people were ready to vaccinate if the vaccination was not free and (ii) asking for owners to pay a nominal fee could be counterproductive [32]. Therefore, to reach dog vaccination coverage goals, the center-piece of rabies elimination strategies, we strongly recommend authorities in charge of rabies control to focus efforts on free-of-charge mass dog vaccination. Providing vaccine through routine visits of veterinarians have been shown to be more effective to reach more remote rural communities than static vaccination points in the Malagasy context [32]. Thus, to reach vaccination coverage targets, awareness campaigns need to be organized simultaneously with free-of-charge mass dog vaccination with high spatial outreach. Communities should be informed on efficacy, safety and importance of dog vaccination, as well as practical modalities of the vaccination campaign.

### Prior awareness and knowledge on rabies

Overall, a high proportion of respondents had prior awareness of rabies (91.9%), both in the remote and central area, suggesting that this was not a consequence of the vaccination campaign organized by Mad Dog Initiative (focused in the urban commune of Moramanga). Such levels are similar to previous findings in Moramanga [8] and in other rural areas in Africa [27,35,37,40,43] and in Asia [47,49]. These results are not surprising given the regular confirmation of human and animal rabies cases in the Moramanga area [14]. Yet, this question should be interpreted with caution as among the 27 respondents who said they did not have prior awareness of rabies, 23 (85%) correctly answered that rabies could be transmitted through a bite. Furthermore, out of the 27, 17 (63%) chose to answer the question if rabies could be transmitted by animals and only 10 (37%) answered that they did not know. Out of the 17 who answered the latter question, 16 (94%) answered that rabies could be transmitted by animals and only one (6%) that rabies could not be transmitted by animals suggesting that the proportion who really guessed what to answer was low. This question on prior awareness of rabies might have been biased because of a reinterpretation of the question, people who had prior awareness of rabies but were not sure of being able to talk of rabies in detail might have answered “no” to the question “are you aware of rabies?”. This may limit our understanding on how to reduce the proportion of people who say they don’t have prior awareness of rabies, a variable which was retained both the knowledge and practice score models (although the association was not statistically significant).

Here 21.0% of respondents (n = 70) in the study mentioned having benefited from rabies awareness campaign, with a larger proportion in the central area (30.0% *vs*. 11.6%, *p*<0.001). Although most of the respondents had benefited from the campaign recently in both areas (mean = 1.6 and 1.3 years, *p =* 0.5), having benefited from an awareness campaign was not significantly associated to better knowledge or practice scores. The qualitative survey showed that acquaintances were the principal source of information on what to do when someone has been bitten, in line with previous results in Moramanga [8] and with other studies in Africa [41,80].

Regarding knowledge on rabies, overall, many aspects of rabies were well-known such as the main species involved virus transmission, main transmission pathways (bites, scratches, contact with saliva on broken skin), main symptoms in animals (drool, aggressivity) and that rabies was fatal. Yet few people knew that ruminants could also transmit rabies (29.4%), as commonly observed in other settings [27,37,51]. Over 90% of respondents thought rabies was curable, but this could be due to an unclear wording of the question (interview bias) as it is not clear whether they were referring to rabies infection before or after symptom onset and if they were referring to PEP as the cure.

Lack of knowledge on the existence of ARTCs and the necessity to seek PEP as soon as possible are probably the knowledge gaps which have the most deleterious impact on the Zero by 30 goal. Indeed, the percentage of respondents who were aware of the existence of the ARTC was dramatically low (14.7%), even among those living less than 5 km away (18.2% *vs*. 11.0% in the remote area, *p =* 0.06). It is crucial to inform the population on the existence and location of ARTC, the availability of free-of-charge vaccine for exposed patients in ARTCs and that PEP should be sought as rapidly as possible as rabies is fatal after the onset of clinical signs. People also need to know that rabies can be transmitted even through superficial wounds and that all dogs can transmit rabies (not just feral ones).

A large majority of respondents (83.5%) thought that rabies could be transmitted by the ingestion of meat or milk from a rabid animal. This is much more than in Ethiopia for example where only 2% and 0.5% of respondents (n = 400) said that rabies could be transmitted through the ingestion of raw meat and raw milk respectively [37]. Raising awareness on the fact that people who have eaten meat from a rabid animal do not need to seek PEP would also be very useful, as this situation occurs regularly in Madagascar and leads to important groups of people visiting the ARTC to seek PEP (when a large number of people from a locality have eaten meat from a rabid zebu for example), and this wastage can lead to vaccine shortage. This wastage is important: in Moramanga, 20% of patients reporting for PEP at the ARTC were classified as low-to-no risk contacts [14].

### Bite victims and bite management practices

The percentage of households in which a member had been bitten (7.5%) was similar to what was observed in Moramanga city in 2011 [8] but much lower than what was observed in Tanzania [27] where approximately the same percentage of households owned dogs, but where household size and the number of dogs per household were greater than what we observed here.

Bite incidence crude estimates were higher in the central area than in the remote area (0.53 *vs*. 0.22 per 100 person-years respectively) despite similar dog-to-human ratios. Reasons explaining this difference should be further explored. Are contacts with dogs more frequent in areas where urban fabric is denser? Are the types and contexts of contacts different? Bite incidence is multifactorial and varies greatly across countries, making it difficult to compare situations. Nevertheless, bite incidence in the central area was very similar to what was observed in Democratic Republic of Congo (5.2 per 1000 person-years) [25], despite a lower dog-to-human ratio (1:37.7) than in Moramanga. A more precise assessment of bite incidence and its spatial heterogeneity, as well as a more thorough understanding of factors driving bite incidence would be useful to better target prevention measures.

Concerning practices after a bite, only 45.5% of respondents considered the wound should be washed, similar to what was observed in Morocco [36]. Superficial wounds were more likely to be disregarded, as observed in other studies [8,76]. Overall, 87.7% would seek medical advice from a doctor or go to a medical center, but only 6.6% stated that the bitten person should seek PEP. The percentage of respondents who mentioned that bite victims should seek PEP was very low, as in Cameroon (Barbosa Costa 2018). The qualitative survey confirmed that PEP was not sought immediately. The care pathway comprised firstly a visit to the dog’s owner to know if the dog was vaccinated and if the owner was willing to cover the costs, and only in a second stage the bite victim would eventually consult a health center, which in turn referred the patient to an ARTC. The willingness to avoid social conflicts within the community was strong: the bite victim usually did not want to confront the owner and create a conflict in the community if the owner did not accept to cover the care costs, in accordance with the Malagasy cultural concept of “fihavanana”, strong social links and moral obligations which entail to take care of others as kins and mutually help each other to guarantee social harmony and unity [81]. The whole process is likely to slow down PEP delivery, possibly reducing the chances of survival. Awareness campaigns should explain the importance of wound washing and of immediately seeking PEP after a bite. Population including dog owners should be aware that PEP is available and free in ARTCs.

KAP studies in Asia and Africa have shown that traditional treatments can be very frequent [28,36,51,82] and can act as barriers to seek medical treatment [41]. In our study, only 0.3% said they would seek traditional treatment after a bite, in line with the 4.5% of respondents who believed that traditional treatments were effective against rabies, much less than what was documented in Ethiopia [35], Morocco [36] or India [51]. As field investigators were clearly identified as linked to conventional medicine, a social desirability or courtesy bias could have impacted these results as suggested by the fact that more respondents reported using traditional treatments when questioned on their practices (11.4% (n = 38) of respondents answered they would treat the wound with oil) than when asked if they would seek traditional treatments in the event of a bite (0.3% reported they would seek this type of treatment). Use of oil was also frequently mentioned in the qualitative study, yet this did not seem to exclude seeking biomedical care in health centers and in the ARTC (since respondents of the qualitative survey were recruited through the ARTC). A malaria anthropology study conducted in Madagascar showed that traditional beliefs are very present in Madagascar but traditional beliefs and biomedical explanations are not mutually exclusive [83]. Another study in Madagascar concluded that too much emphasis had been given to traditional practices and social norms as barriers to changing attitudes and behaviors towards health [84]. Although some cultural practices remained strong, in many cases, the obstacles were rather of the order of supply, distance and cost of services rather than cultural beliefs and traditions according to that study [84].

Concerning the fate of the biting dog, the consequences of a bite varied between two extremes: either nothing happened to the dog, either it was killed. When a person was bitten by a dog, 24.9% thought the dog should be killed, but if a known dog had bitten someone, then 48.8% would consider killing it. Similar results were found in the qualitative approach: the dog was killed mainly if the owner did it himself (as in four of the five cases where the dog was killed). Indeed, if the dog was known, the person could more easily decide to kill it in order to maintain social cohesion, but when the dog was not known, then they left the owners take their responsibilities and decide what to do with the dog. Thus, it showed the importance of social pressure not to disrupt relations in the community and also the weak attachment to dogs.

Only 5.7% mentioned that the dog should be restricted and placed under observation, and this finding was confirmed in the qualitative study. Awareness campaigns should aim to promote this practice, adapting it to the context, which, in poor and/or remote rural households might mean isolating the dog at home due to cost and low access to veterinarians. Alongside to awareness campaigns, increasing access to veterinarians and animal health workers would of course also contribute to improve practices, surveillance and animal health in general.

### Factors associated to knowledge and practices

Several factors impacting knowledge and practices towards rabies in the community of Moramanga were identified. Respondents with high socio-economic status (OR = 2.08) and living in the central area within 5 km of the ARTC (OR = 1.91) were significantly likely to be more knowledgeable. Two further variables improved the model although their associations with the knowledge score were not statistically significant: having prior awareness of rabies (OR = 1.85) and having owned a dog in the previous 5 years (OR = 1.49). Respondents living in the central area (OR = 4.78) and who were aware of the ARTC’s existence (OR = 2.29) were significantly more likely to have better practices. Prior awareness of rabies (OR = 2.21) and having a high socio-economic status (OR = 1.47) contributed to improve the model although their associations with the practice score were not statistically significant. Respondents with a higher knowledge score also had a significantly higher practice score. Overall, results show that prior awareness on rabies and the ARTC are key to increase appropriate health seeking practices and that remote rural areas and households with low socio-economic status need to be specifically targeted.

Distance to the ARTC impacted both knowledge and practices despite testing only a limited range of distances as households from the remote area were all less than 30 km away from the ARTC. Knowing that 36% of the population are estimated to live at least 3 hours away from the closest ARTC [12] and that some households may be located 50 to 100 km from an ARTC question as to what are the KAP in households which are located further than 30 km from the closest ARTC. A KAP study targeting these very remote communities would help fill this important knowledge gap as these populations may be even less knowledgeable towards rabies prevention and at greater risk of not seeking PEP.

The socio-economic status and level of education are well-known factors associated with better knowledge and practices in several KAP studies [26,33,51]. Our study confirms the association of socio-economic factors with the knowledge score but the association was not statistically significant with the practice score. This might partly be explained by the fact that PEP is free-of-charge for exposed patients in Madagascar. Unfortunately, individual information on the level of education was not collected in this study. The MHURAM project showed that in the urban commune of Moramanga, 2.4% of people never went to school, 31.6% had gone to primary school, 59.0% to middle school and 7.0% to high school or to a professional school whereas in the rural communes, 11% never went to school, 60.5% had gone to primary school, 27.7% to middle school and 0.8% to high school or professional school [63]. As level of education was lower in the rural communes, it would be important to disentangle the effects of the two to know whether part of the effect of living in the remote rural area on the decrease of knowledge and practice scores, observed in our models, is due to more limited access to education. Awareness on the disease and on PEP might in turn be associated with a higher level of education, whether they act as proxies of the latter remains yet to be fully explored.

## Conclusions and recommendations

This qualitative and quantitative community-based survey on knowledge, attitudes and practices on rabies in Moramanga community showed that the knowledge in the community was rather satisfying concerning the role of dogs in transmission but limited knowledge and unfavorable practices were found in terms of appropriate first aid measures, recourse to medical care and animal management. A key finding of this study was the negative impact of living in more rural communes further from the ARTC on both scores. This suggests that awareness campaigns should be particularly strengthened in remote rural areas and their coverage carefully monitored to make sure they reach the greatest number of households. Another key finding was that being aware of the ARTC’s existence was associated with better practices, suggesting that awareness campaigns should clearly inform populations on the existence, location and availability of free-of charge PEP in the ARTCs, alongside to the necessity to seek PEP when exposed. Finally, within both areas, households with lower socio-economic status should be targeted to improve knowledge on rabies.

This survey also collected some key figures for rabies control such as the percentage of households owning dogs, dog-to-human ratios, the percentage of vaccinated dogs and bite incidence which are important when designing mass dog vaccination campaigns. It also showed that several factors impacted the steps of the care pathway: the severity of the wound, how the biting dog was perceived and to which category it belonged, whether its owner said it was vaccinated (whether he had a vaccination certificate or not) and whether the owner agreed to cover costs.

Overall, awareness campaigns should focus on (i) the necessity to wash the wound with soap and seek PEP as soon as possible after a bite, and (ii) the availability of free-of-charge PEP for exposed patients in ARTCs and their locations. They should encourage seeking care after the bite of any category of dog, and even if the wound is superficial. They should also encourage owners of biting dogs to confine (or attach) and monitor the health and behavior of biting dogs at home if they are unable to seek the services of animal health care workers. Although bite incidence was lower, remote areas had more limited knowledge, poorer practices on rabies and extremely low dog vaccination coverage, so awareness and vaccination campaigns should be carefully designed so as to make sure more remote rural populations are reached.

Finally, if awareness campaigns are likely to succeed in improving access to ARTCs in Madagascar, contributing to the Zero by 30 goal, their impact on dog vaccination seems more uncertain because vaccination costs and low access to animal health practitioners are important barriers in rural communities. Therefore, to reach dog vaccination coverage goals enabling rabies elimination, we strongly recommend combining awareness campaigns with free-of-charge mass dog vaccination with high spatial outreach.

## Supporting information

S1 Table Socio-economic score assessment in the remote and central areas. (XLSX)

S2 Table Reasons for not vaccinating dogs. (XLSX)

S3 Table Comparison of knowledge on rabies in the remote and central areas. (XLSX)

S4 Table Variables associated to the knowledge score in the bivariate analysis. (XLSX)

S5 Table Knowledge score model selection. (XLSX)

S6 Table Spatial autocorrelation of the knowledge and practice scores and of the residues of the knowledge and practice models. (DOCX)

S7 Table Characteristics and practices of bitten individuals. (XLSX)

S8 Table Comparison of practices towards rabies in the remote and central areas. (XLSX)

S9 Table Comparison of practices carried out by a bitten member of the household to practices that the respondent thought should be carried out in the event of a bite. (XLSX)

S10 Table Variables associated to the practice score in the bivariate analysis. (XLSX)

S11 Table Practice score model selection. (XLSX)

S12 Table Dataset. (XLSX)
