# Supplementary material for: Mixed methods to evaluate knowledge, attitudes and practices (KAP) towards rabies in central and remote communities of Moramanga district, Madagascar
Source: PLoS Negl Trop Dis. 2024 Mar 29;18(3):e0012064. doi: 10.1371/journal.pntd.0012064 (PMC11006160; doi:10.1371/journal.pntd.0012064)
Supplement: S6 Table — (DOCX) [file pntd.0012064.s006.docx]

**S6 Table A: Spatial autocorrelation analysis of the knowledge score**

| **Entire area** | **Central Area** | **Remote Area** |
| --- | --- | --- |
| \| **Moran’s global I** \| \| \| --- \| --- \| \| **Moran Index :** \| 0.117312 \| \| **Expected Index :** \| -0.003012 \| \| **Variance :** \| 0.001792 \| \| **Z score :** \| 2.842336 \| \| ***P* value :** \| 0.004478 \| | \| **Moran’s global I** \| \| \| --- \| --- \| \| **Moran Index :** \| 0.019080 \| \| **Expected Index :** \| -0.005952 \| \| **Variance :** \| 0.001818 \| \| **Z score :** \| 0.587073 \| \| ***P* value :** \| 0.557155 \| | \| **Moran’s global I** \| \| \| --- \| --- \| \| **Moran Index :** \| 0.202345 \| \| **Expected Index :** \| -0.006135 \| \| **Variance :** \| 0.015755 \| \| **Z score :** \| 1.660934 \| \| ***P* value :** \| 0.096727 \| |
| \| Dataset \| \| \| --- \| --- \| \| **File:** \| KS2024md4 \| \| **Entry:** \| KSCORE \| \| **Conceptualisation:** \| INVERSE_DISTANCE \| \| **Distance:** \| EUCLIDEAN \| \| **Distance threshold:** \| 3015.5233 Meters \| | Dataset   \| **File:** \| KS2024md4_CENTRAL \| \| --- \| --- \| \| **Entry:** \| KSCORE \| \| **Conceptualisation:** \| INVERSE_DISTANCE \| \| **Distance:** \| EUCLIDEAN \| \| **Distance threshold :** \| 2375.4513 Meters \| | Dataset   \| **File:** \| KS2024md4_REMOTE \| \| --- \| --- \| \| **Entry:** \| KSCORE \| \| **Conceptualisation:** \| INVERSE_DISTANCE \| \| **Distance:** \| EUCLIDEAN \| \| **Distance threshold:** \| 3015.5233 Meters \| |

**S6 Table B: Spatial autocorrelation analysis of the residues of the knowledge score model**

| **Entire area** | **Central Area** | **Remote Area** |
| --- | --- | --- |
| \| **Moran’s global I** \| \| \| --- \| --- \| \| **Moran Index :** \| 0,008105 \| \| **Expected Index :** \| -0,003012 \| \| **Variance :** \| 0,001827 \| \| **Z score :** \| 0,260079 \| \| ***P* value :** \| 0,794803 \| | \| **Moran’s global I** \| \| \| --- \| --- \| \| **Moran Index :** \| -0,034106 \| \| **Expected Index :** \| -0,005952 \| \| **Variance :** \| 0,001872 \| \| **Z score :** \| -0,650702 \| \| ***P* value :** \| 0,515239 \| | \| **Moran’s global I** \| \| \| --- \| --- \| \| **Moran Index :** \| 0,163481 \| \| **Expected Index :** \| -0,006135 \| \| **Variance :** \| 0,016288 \| \| **Z score :** \| 1,329006 \| \| ***P* value :** \| 0,183846 \| |
| \| Dataset \| \| \| --- \| --- \| \| **File:** \| KS2024md4 \| \| **Entry:** \| RES_MD4 \| \| **Conceptualisation:** \| INVERSE_DISTANCE \| \| **Distance:** \| EUCLIDEAN \| \| **Distance threshold:** \| 3015,5233 Meters \| | Dataset   \| **File:** \| KS2024md4_CENTRAL \| \| --- \| --- \| \| **Entry:** \| RES_MD4 \| \| **Conceptualisation:** \| INVERSE_DISTANCE \| \| **Distance:** \| EUCLIDEAN \| \| **Distance threshold:** \| 2375.4513 Meters \| | Dataset   \| **File:** \| KS2024md4_REMOTE \| \| --- \| --- \| \| **Entry:** \| RES_MD4 \| \| **Conceptualisation:** \| INVERSE_DISTANCE \| \| **Distance:** \| EUCLIDEAN \| \| **Distance threshold:** \| 3015.5233 Meters \| |

**S6 Table C: Spatial autocorrelation analysis of the practice score**

| **Entire area** | **Central Area** | **Remote Area** |
| --- | --- | --- |
| \| **Moran’s global I** \| \| \| --- \| --- \| \| **Moran Index :** \| 0.225893 \| \| **Expected Index :** \| -0.003058 \| \| **Variance :** \| 0.001864 \| \| **Z score :** \| 5.302648 \| \| ***P* value :** \| 0.000000 \| | \| **Moran’s global I** \| \| \| --- \| --- \| \| **Moran Index :** \| 0.069476 \| \| **Expected Index :** \| -0.006061 \| \| **Variance :** \| 0.001913 \| \| **Z score :** \| 1.726911 \| \| ***P* value :** \| 0.084184 \| | \| **Moran’s global I** \| \| \| --- \| --- \| \| **Moran Index :** \| 0,052944 \| \| **Expected Index :** \| -0,006211 \| \| **Variance :** \| 0,015798 \| \| **Z score :** \| 0,470636 \| \| ***P* value :** \| 0,637901 \| |
| \| Dataset \| \| \| --- \| --- \| \| **File:** \| PS2024v3 \| \| **Entry:** \| PSCORE \| \| **Conceptualisation:** \| INVERSE_DISTANCE \| \| **Distance:** \| EUCLIDEAN \| \| **Distance threshold :** \| 3015.5233 Meters \| | \| Dataset \| \| \| --- \| --- \| \| **File:** \| PS2024v3Central \| \| **Entry:** \| PSCORE \| \| **Conceptualisation:** \| INVERSE_DISTANCE \| \| **Distance:** \| EUCLIDEAN \| \| **Distance threshold:** \| 2375.4513 Meters \| | \| Dataset \| \| \| --- \| --- \| \| **File:** \| PS2024v3Remote2 \| \| **Entry:** \| PSCORE \| \| **Conceptualisation:** \| INVERSE_DISTANCE \| \| **Distance:** \| EUCLIDEAN \| \| **Distance threshold:** \| 3015,5233 Meters \| |

**S6 Table D: Spatial autocorrelation analysis of the residues of the practice score model**

| **Entire area** | **Central Area** | **Remote Area** |
| --- | --- | --- |
| \| **Moran’s global I** \| \| \| --- \| --- \| \| **Moran Index :** \| 0,028184 \| \| **Expected Index :** \| -0,003058 \| \| **Variance :** \| 0,001879 \| \| **Z score :** \| 0,720809 \| \| ***P* value :** \| 0,471027 \| | \| **Moran’s global I** \| \| \| --- \| --- \| \| **Moran Index :** \| 0,016988 \| \| **Expected Index :** \| -0,006061 \| \| **Variance :** \| 0,001929 \| \| **Z score :** \| 0,524781 \| \| ***P* value :** \| 0,599736 \| | \| **Moran’s global I** \| \| \| --- \| --- \| \| **Moran Index :** \| 0,039131 \| \| **Expected Index :** \| -0,006211 \| \| **Variance :** \| 0,016409 \| \| **Z score :** \| 0,353967 \| \| ***P* value :** \| 0,723364 \| |
| \| Dataset \| \| \| --- \| --- \| \| **File:** \| PS2024v3 \| \| **Entry:** \| RES_PSM4B \| \| **Conceptualisation:** \| INVERSE_DISTANCE \| \| **Distance:** \| EUCLIDEAN \| \| **Distance threshold :** \| 3015.5233 Meters \| | \| Dataset \| \| \| --- \| --- \| \| **File:** \| PS2024v3Central \| \| **Entry:** \| RES_PSM4B \| \| **Conceptualisation:** \| INVERSE_DISTANCE \| \| **Distance:** \| EUCLIDEAN \| \| **Distance threshold:** \| 2375.4513 Meters \| | \| Dataset \| \| \| --- \| --- \| \| **File:** \| PS2024v3Remote2 \| \| **Entry:** \| RES_PSM4B \| \| **Conceptualisation:** \| INVERSE_DISTANCE \| \| **Distance:** \| EUCLIDEAN \| \| **Distance threshold:** \| 3015,5233 Meters \| |
